# Comparison of postoperative CT- and preoperative MRI-based breast tumor bed contours in prone position for radiotherapy after breast-conserving surgery

**DOI:** 10.1007/s00330-020-07085-0

**Published:** 2020-08-01

**Authors:** Yinping Dong, Yang Liu, Jinhu Chen, Wanhu Li, Yongqing Li, Qian Zhao, Yiru Chen, Wei Huang

**Affiliations:** 1 School of Medicine and Life Sciences, University of Jinan-Shandong Academy of Medical Sciences, Jinan, China; 2 Department of Radiation Oncology, Shandong Cancer Hospital and Institute, Shandong First Medical University and Shandong Academy of Medical Sciences, 440 Jiyan Road, Jinan, 250117 Shandong China; 3 Lung Cancer Center, West China Hospital, Sichuan University, Chengdu, Sichuan China; 4 Department of Radiology, Shandong Cancer Hospital and Institute, Shandong First Medical University and Shandong Academy of Medical Sciences, Jinan, China; 5 Department of Surgical Ward 1, Shandong Cancer Hospital and Institute, Shandong First Medical University and Shandong Academy of Medical Sciences, Jinan, China

**Keywords:** Breast cancer, Magnetic resonance imaging, Radiotherapy

## Abstract

**Objectives:**

To compare the target volume of tumor bed defined by postoperative computed tomography (post-CT) in prone position registered with or without preoperative magnetic resonance imaging (pre-MRI).

**Methods:**

A total of 22 patients were included with early-stage breast invasive ductal cancer, who have undergone breast-conservative surgery and received the pre-MRI and post-CT in prone position. The MRI sequences (T1W, T2W, T2W-SPAIR, DWI, dyn-eTHRIVE, sdyn-eTHRIVE) were delineated and manually registered to CT, respectively. The clinical target volumes (CTVs) and planning target volumes (PTVs) were contoured on CT and different MRI sequences, respectively. Differences were measured in terms of consistence index (CI), dice coefficient (DC), geographical miss index (GMI), and normal tissue index (NTI).

**Results:**

The differences of delineation volumes among CT and MRIs were significant, both in the CTVs (*p* = 0.035) and PTVs (*p* < 0.001). The values of CI and DC for sdyn-eTHRIVE registration to CT were the largest among all MRI sequences, but GMI and NTI were the smallest. No obvious linear correlation (*p* > 0.05) between the CI derived from the registration of CT and sdyn-eTHRIVE of CTV with the breast volume, the cavity visualization score (CVS) of CT, time interval from surgery to CT simulation, the maximum diameter of the intraoperative mass, and the number of titanium clips, respectively.

**Conclusions:**

The CTVs and PTVs in MRI sequences were all smaller than those in CT. The pre-MRI, especially the sdyn-eTHRIVE, could be used to optimize the post-CT-based target delineation of breast cancer.

**Key Points:**

*• Registered pre-MRI to post-CT in order to improve the accuracy of target volume delineation of breast cancer.*

*• The CTVs and PTVs in MRI sequences were all smaller than those in CT.*

*• The sdyn-eTHRIVE of pre-MRIs may be a better choice to improve the delineation of CT-based CTV and PTV.*

**Electronic supplementary material:**

The online version of this article (10.1007/s00330-020-07085-0) contains supplementary material, which is available to authorized users.

## Introduction

Boost to the tumor bed (TB) following whole-breast radiotherapy could improve the local recurrence rates in patients with conservative breast cancer [1–3]. However, there was neither precise definition of TB nor optimal imaging modalities provided for the target volume delineation. Currently, radiation oncologists usually outline the TB in a variety of ways, but the position and the shape of the TB could not be determined accurately. All these elements, including the patient’s surgical records, surgical scar [4], postoperative palpation changes, clips placed around the bed [5, 6], and presurgical or postoperative breast imaging examinations, such as mammography, ultrasonography, computed tomography (CT) imaging, and magnetic resonance imaging (MRI), were all taken into account. Among them, CT imaging, as a popular method of the whole-breast delineation and boost volume definition by using clips and postoperative seroma to define TB, could not clearly define the TB because of its limited soft tissue contrast, leading to the obvious variations between different observers [7–10].

Because of its superb soft tissue contrast [11, 12] and better definition of TB [13], MRI, especially the postoperative MRI (post-MRI), has been incorporated into target volume definition under the premise of CT-positioned and CT-determined TB, as well, the co-registration of the MRI and CT imaging modalities could result in improved inter-observer concordance in delineation [7, 14]. MRI scans are obtained in prone position, which could reduce the volume of lung or heart exposed to later irradiation [15], especially for those with large and pendulous breast size. In order to improve the accuracy of the TB delineation, our previous study delineated the lumpectomy cavity (LC) volume on the basis of the registration imaging of post-MRI and postoperative CT (post-CT) in prone position, which pointed out that MRI could improve the visibility of LCs compared with CT, and therein the MRI-STIR sequence showed the highest visibility [16].

However, although the post-MRI could clearly manifest the location and the contour of the postoperative seroma, the seroma would shrink and even disappear over time [17]. So the image of post-MRI could not be considered as the representative of TB virtually because of its impaired visibility to clips [18]. Nowadays, more and more studies have registered preoperative imaging (pre-MRI and/or pre-CT) to post-CT in order to improve the accuracy of target volume delineation. In a study conducted by den Hartogh et al, TBs of breast cancer were delineated on pre-CT and pre-MRI, and the consistency in target volume delineation was evaluated. They found that TB delineation on preoperative imaging could increase the consistency among observers, and pre-MRI was significant for the detection of tumor [19]. At the same time, pre-MRI could specifically show the preoperative details of the mass. Therefore, we performed this study aimed to compare the clinical target volumes (CTVs) and planning target volumes (PTVs) defined by pre-MRI and postoperative prone CT in order to search for the best settings of the pre-MRI, which in turn optimizes the TB delineation.

## Materials and methods

### Cohort of patients

We collected clinical records and imaging data (including pre-MRI and postoperative planning CT data) of patients with early-stage breast cancer between June 2016 and June 2018, who have undergone breast conservative surgery and were pathologically diagnosed with invasive ductal carcinoma (pathologically T1-2; N0-1; M0) with 3–9 titanium clips intraoperatively placed within the LCs and scheduled to adjuvant radiotherapy. Patients who received neoadjuvant chemotherapy or endocrinotherapy with oncoplastic surgery or harboring contraindication or intolerant for MRI were excluded. This study was approved by the institutional review board of Shandong Cancer Hospital and Institute, and all participants signed the informed consents.

### Pre-MRI and post-CT image acquisition

The pre-MRI was performed with a mean of 2.3 days (range, 1–7) before the breast-conserving surgery at our institution, for a standard imaging protocol, which includes six sequences: T1W, T2W, T2W-SPAIR (T2-weighted spectral attenuated inversion recovery), DWI (diffusion-weighted imaging), dyn-eTHRIVE (dynamic-enhanced T1 high-resolution isotropic volume excitation), and sdyn-eTHRIVE (subtraction of dynamic-enhanced T1 high-resolution isotropic volume excitation). The images of sdyn-eTHRIVE, named by Philips, were subtracted with the original images prior to the administration of contrast agents from enhanced images, which could reduce the influence of non-tumor-enhanced tissue or normal gland tissue on the tumor mass judgment. The CT scan was acquired after the operation about 99 days at average (range: 26–204 days) in prone position by using a specific immobilization device (Supplementary Figure 1). This process was undertaken on a big-bore CT scanner (Brilliance, Philips, Neth.) and scanned from the cricothyroid membrane to the lower edge of the liver with 3-mm interslice thickness. The field of view (FOV) of CT is 450 mm, and the pixel value is 512 × 512. Consistent with pre-MRI for better registration, the slice thickness of post-CT was reconstructed to 4 mm. The surgical scars were labeled by metal wires to help determine the rough location of TB. The MRI images were obtained in routine position (prone) with the use of a 3.0-T MR scanner with a 60-cm bore (Achieva 3.0T, Philips Healthcare) with special double-acupoint 16-channel breast surface coil (Supplementary Figure 2). Among this, with no slice gap, the T1W, T2W, T2W-SPAIR, and DWI were collected with a slice thickness of 4 mm, but the slice thickness of dyn-eTHRIVE was 1 mm, so did the sdyn-eTHRIVE. The slice thickness of dyn-eTHRIVE and sdyn-eTHRIVE was reconstructed to 4 mm to maintain consistency among all sequences. The parameters of T1W, T2W, T2W-SPAIR, DWI, and dyn-eTHRIVE, such as echo time (TE), repetition time (TR), FOV, pixel values, and other corresponding parameters, are listed in Table 1. As well, with *b*-factors of 0 and 800 s/mm^2^ in one scan of DWI, the images with *b* = 800 s/mm^2^ were selected as representatives for DWI, which shows a clearer outline of the tumor lump on most patients, notwithstanding at the expense of some pixels. The dyn-eTHRIVE is characterized as eight sequences (1–8) obtained by a phase before the injection of the contrast-enhancing agent plus 7 consecutive repetition phases, that is, the continuous uninterrupted scanning performed 7 times after the injection of the contrast agent immediately. We used a 20-mL high-pressure syringe of bolus-injected gadopentetate dimeglumine (0.1 mmol/kg) at 3 mL/s followed by a 15-mL saline flush to get the enhancing images. Considered the characteristics of early enhancement of invasive ductal carcinoma [20], combined with the clinical recommendations of the MRI radiologists, we delineated the target volume on the image of 2, 3, 4, and 5 phases and select the 3 phase image as the representative image of the dyn-eTHRIVE sequence. Likewise, the phase before the injection of the contrast-enhancing agent subtracted from the 3 phases of dyn-eTHRIVE was chosen to delineate the sdyn-eTHRIVE.

**Table 1:** Parameters of MRI sequences. T1W, T2W, T2W-SPAIR, and DWI are all two-dimensional images, whose pixel values include AP and RL directions. And the dyn-eTHRIVE is a kind of three-dimensional volumetric image, and its pixel value includes three directions: AP, RL, FH

Parameters	TE (ms)	TR (ms)	NSA	FOV (mm)	Matrix	Pixel value (mm) (AP × RL × FH)	Scan time	*b* value (s/mm^2^)
T1W	10	495	1	340	340 × 271	1.0 × 1.25	2′ 53″ 1	—
T2W	120	4213	1	340	452 × 332	0.75 × 1.0	2′ 14″ 8	—
T2W-SPAIR	60	4216	1	340	340 × 267	1.0 × 1.25	1′ 41″ 2	—
DWI	51	7099	2	340	120 × 120	2.8 × 2.8	1′ 46″ 5	800
dyn-eTHRIVE	Shortest	Shortest	1	340	340 × 340	1.0 × 1.0 × 1.0	7′ 4″ 0 (0′ 53″ 0 × 8)	—

*TE*, echo time; *TR*, repetition time; *NSA*, number of signal averaged; *FOV*, field of view; *AP*, anterior/posterior direction; *RL*, right/left direction; *FH*, foot/head direction

### Image processing

The MRI images were transferred to Eclipses’ Treatment Planning Systems (TPSs) for registration and delineation. All MRI sequences were respectively conversed to head-first prone status to register with CT on the basis of rigid registration accompanied with manual alignment registration by application of titanium clips, the lump, and various anatomic features including the nipple, sternum, and vertebra, especially the mammary glands. Focus laid on the mammary gland concordance. Next, the concordance between the TB determined by the clips and the primary lump was also taken into account, equally, lying in accurate registration, which contributes to better comparison of consistency parameters.

### Structure delineation

**Fig. 1:**
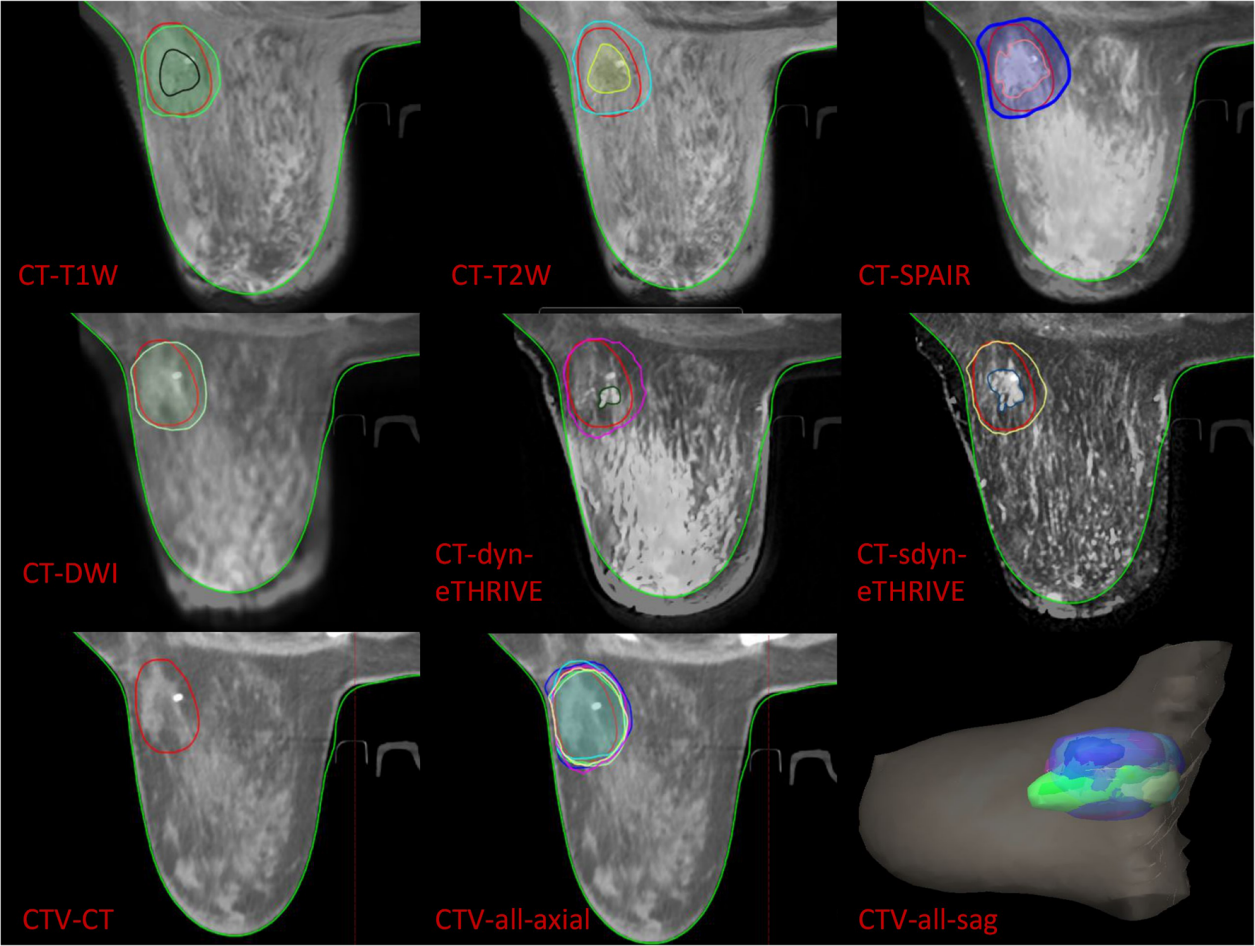
The delineation and registration of CTVs

**Fig. 2:**
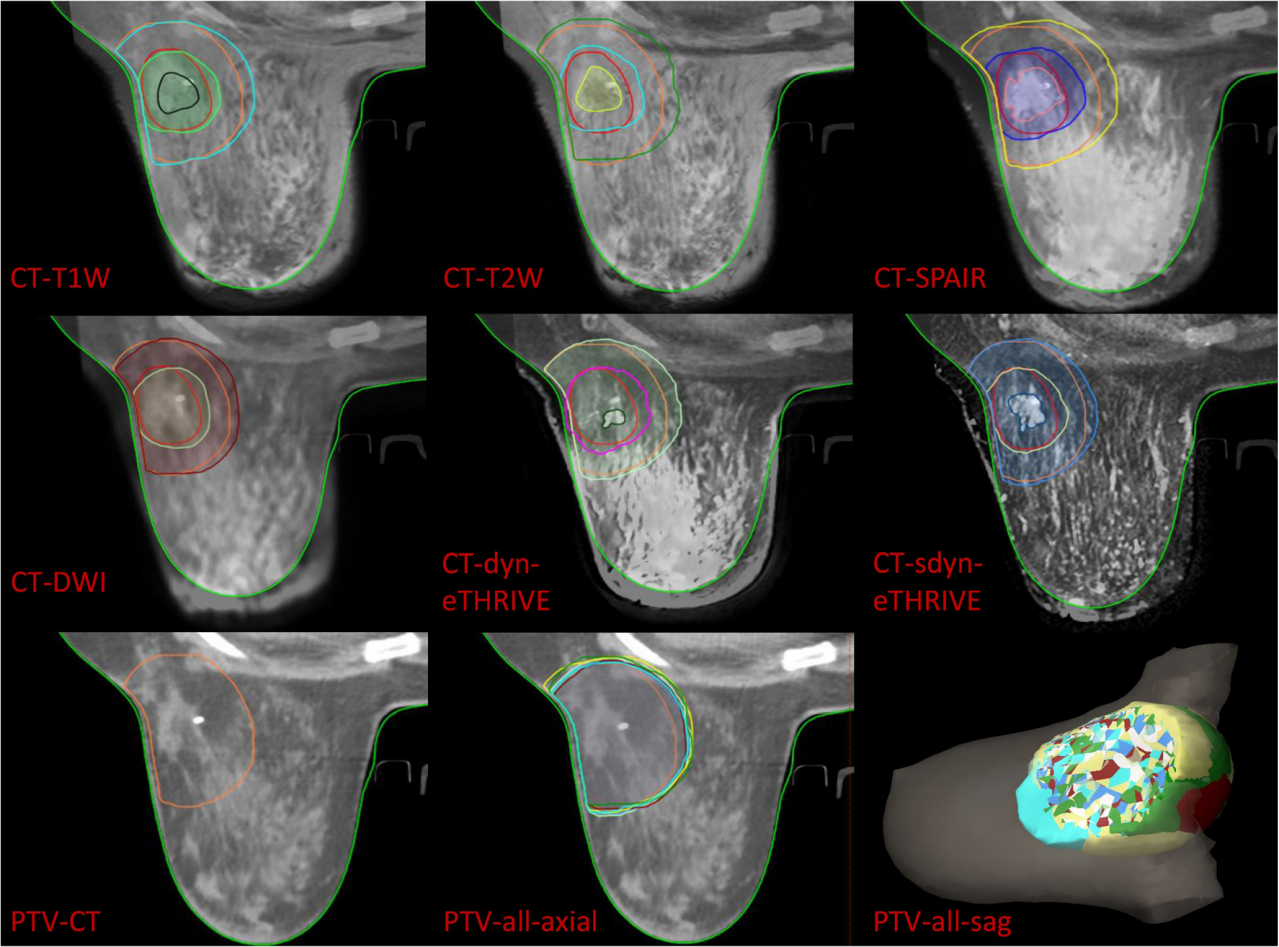
The delineation and registration of PTVs

**Fig. 3:**
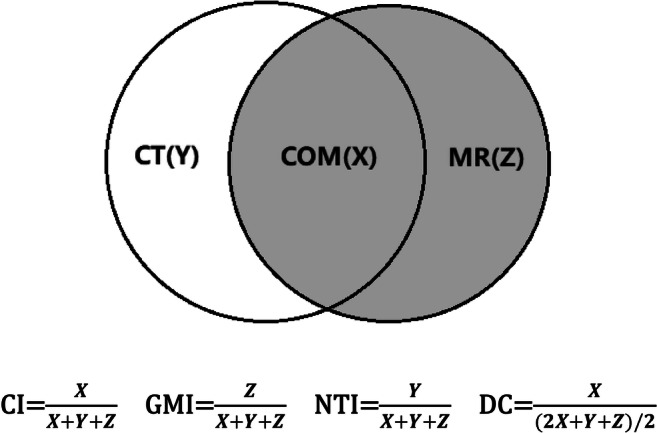
Parameter calculation. Each MRI sequence was scheduled to register to CT, respectively. The volumes of *X*, *Y*, and *Z* were acquired from the treatment planning system. The *X* represents the overlapping target volume between CT and MRI. The *Y* represents the target volume of CT minus the volume of *X*, and the *Z* refers to the target volume of MRI minus the volume of *X*. The consistence index (CI) and dice coefficient (DC) of CTVs and PTVs between CT and each MRI sequence were measured to quantify the extent of overlap of two volumes, and the geographical miss index (GMI) and normal tissue index (NTI) were considered as non-conformity parameters between different imaging modalities. The CI, DC, GMI, and NTI were calculated according to the above formula

The TB delineation of CT and all MRI sequences was entirely performed from only a single modality by an experienced radiation oncologist to avoid inter-observer variation. Meanwhile, it was verified by another oncologist according to the criterion of the Radiation Therapy Oncology Group (RTOG) [9]. The whole breast is outlined on the CT to calculate the volume. The clips, seroma, and surgical scars were applied to guide the CT-based contours for CTVs, while the tumor as visualized on MRI was defined as the standard gross target volume (GTV) for MRI-based delineations. The target volume contours (CTV-CT and GTV-T1, GTV-T2, GTV-T2-SPAIR, GTV-DWI, GTV-dyn-eTHRIVE, and GTV-sdyn-eTHRIVE) were performed on the axial slices; simultaneously, sagittally and coronally reconstructed images could be used to verify the delineations. The corresponding CTV-MRI was obtained respectively by extrapolating 1.0 cm of the GTV-MRI based on the MRI sequence according to the surgeon’s clinical experience and the expert consensus (Fig. 1). And various PTVs were defined as unified margins of 15 mm expanded based on the corresponding TBs, and trimmed 5 mm from the skin to the breast-chest wall interface (Fig. 2). Each MRI sequence was scheduled to register to CT, respectively. The consistence index (CI) and dice coefficient (DC) of CTVs and PTVs between CT and each MRI sequence were measured to quantify the extent of overlap of two volumes; the geographical miss index (GMI) and normal tissue index (NTI) were considered as non-conformity parameters between different imaging modalities. DC = 1 represents perfect overlap, while DC = 0 represents no overlap. The specific calculation of these parameters is shown in Fig. 3. The cavity visualization score (CVS) of CT was also evaluated. CVS = 1 means that the cavity was not visualized; CVS = 2 means that the cavity was visualized with indistinct margins; CVS = 3 means that the cavity was visualized with some distinct margins and heterogeneous appearance; CVS = 4 means that the cavity had distinct margins in the majority of LC with mild heterogeneity; and CVS = 5 means that the cavity has distinct margins in the entire LC with a homogeneous appearance [21].

### Statistical methods

The data were analyzed with SPSS software (version 19.0). Quantitative data were expressed as mean ± standard deviation (SD). The two-way analysis of variance (ANOVA) was used to compare the differences of volumes of delineation among all sequences, including CT and all MRI sequences. Dunnett’s test followed ANOVA to assess the difference of volumes of delineation between each MRI sequence and CT. Similarly, the differences between different registrations, that is, different MRIs registered to CT under the circumstances with the same index, were also analyzed by two-way ANOVA. Then, Dunnett’s tests were performed subsequently to compare the difference between sdyn-eTHRIVE-CT and other MRI-CT for the parameters with statistical significance of two-way ANOVA. Linear regression analysis was used for evaluating correlations between the CI, which are derived from the registration of CT and sdyn-eTHRIVE of CTV, with the breast volume, the CVS of CT, the time interval from surgery to CT simulation, the maximum diameter of the intraoperative mass, and the number of titanium clips, respectively. A *p* value of less than 0.05 was considered significant.

## Results

### Patients’ clinical characteristics and delineation volume comparison

In total, the pre-MRI and postoperative planning CT data of 81 patients were acquired and 22 of these (27.2%) were eligible for analysis. A total of 59 patients (72.8%) were excluded due to not fulfilling the specific inclusion criteria, incomplete clinical records, or unclear imaging data. The detailed data of the enrolled 22 patients are listed in Table 2. For the entire group, the mean ± SD values of the CTVs were 37.350 ± 13.699, 35.255 ± 14.429, 35.827 ± 15.607, 36.891 ± 14.739, 34.086 ± 13.290, 34.191 ± 12.171, and 34.032 ± 12.422 cm^3^ for CT, T1W, T2W, T2W-SPAIR, DWI, dyn-eTHRIVE, and sdyn-eTHRIVE, respectively. For PTVs, the mean ± SD values were 181.246 ± 42.576, 170.023 ± 42.827, 171.082 ± 44.788, 173.564 ± 42.888, 166.641 ± 40.205, 165.873 ± 37.476, and 164.014 ± 37.787 cm^3^ for CT and abovementioned MRI sequences, respectively. The CTVs and PTVs in MRI sequences were all smaller than those in CT. The differences of delineation volumes between all sequences (CT and MRI) were significant, both in the CTVs (*F* = 2.354, *p* = 0.035) and PTVs (*F* = 6.464, *p* < 0.01). Specifically, in CTVs, only the volume of delineation in sdyn-eTHRIVE was significantly different from that in CT (*p* = 0.047). As for PTV delineation, volume in each MRI sequence was significantly different (*p* < 0.05) from that in CT, except the T2W-SPAIR (*p* = 0.088). Therefore, when compared with other MRI sequences, the CTVs and PTVs of sdyn-eTHRIVE are effectively distinguished from the corresponding target volume of CT, as seen in Table 3.

**Table 2:** Clinical characteristics of the included 22 patients

Characteristic	No. of patients
Median age (range)	45 years old (36–63 years old)
Pathologic T stage	
Tis	1
T1b	16
T1c	2
T2	3
FIGO stage	
0	1
I	14
II	7
Histologic grade	
I	2
II	16
III	4
Location	
Right	9
Left	13
No. of surgical clips, mean (range)	5 (3–9)
Time interval from preoperative MRI scanning to surgery (days), median (range)	1 (1–7 days)
Time interval from surgery to CT simulation (days), median (range)	93 (26–204 days)
Breast size	
> 1200 cm^3^	3
800 cm^3^~1200 cm^3^	7
< 800 cm^3^	12
Mean ± SD	795.95 ± 342.55
Median tumor size (in greatest dimension) on MRI at diagnosis (range)	1.5 cm (0.9–3.0 cm)
Median tumor size (in greatest dimension) resected during surgery (range)	1.5 cm (0.8–4.0 cm)

*CT*, computed tomography; *MRI*, magnetic resonance imaging

**Table 3:** Volumes of delineation between CT and MRI

Volume	CT	T1W	T2W	T2W-SPAIR	DWI	dyn-eTHRIVE	sdyn-eTHRIVE	*F*	*p* value^a^
CTV	37.350 ± 13.699	35.255 ± 14.429	35.827 ± 15.607	36.891 ± 14.739	34.086 ± 13.290	34.191 ± 12.171	34.032 ± 12.422	2.354	0.035
	*p* value^b^	0.368	0.682	0.999	0.053	0.065	0.047		
PTV	181.246 ± 42.576	170.023 ± 42.827	171.082 ± 44.788	173.564 ± 42.888	166.641 ± 40.205	165.873 ± 37.476	164.014 ± 37.787	6.464	5.795 × 10^−6^
	*p* value^b^	0.004	0.011	0.088	8.64 × 10^−5^	3.28 × 10^−5^	2.76 × 10^−6^		

^a^Data were calculated by two-way analysis of variance (ANOVA)

^b^Data were calculated by Dunnett’s test to assess the difference between CT and MRI sequences

### Consistence of CTVs

The mean and SD of the consistent parameters between CT and each MRI sequence about CTV are listed in Table 4. With CI compared, there was a significant difference (*p* < 0.05) among different registrations (different MRI sequences registered to CT), as well can be seen in DC and GMI. Specifically, as for CI and DC, each MRI registration to CT was significantly different (all *p* < 0.05) from CT-sdyn-eTHRIVE, except the CT-dyn-eTHRIVE. And significant difference in the value of GMI could only be found between CT-T2W-SPAIR and CT-sdyn-eTHRIVE (*p* = 0.001). However, with the NTI compared, there was no significant difference (*p* > 0.05) among different registrations, meanwhile the values of the conformity parameters CI and DC of CT-sdyn-eTHRIVE were the largest among all MRI registrations to CT, and the non-conformity parameters GMI and NTI were the smallest, which indicated that the overlap between the two sequences is the most.

**Table 4:** Consistent parameters of registrations between CT and different MRI sequences in CTVs

Registrations	CT-sdyn-eTHRIVE	CT-T1	CT-T2	CT-T2W-SPAIR	CT-DWI	CT-dyn-eTHRIVE	*F*	*p* value^a^
CI	0.743 ± 0.046	0.710 ± 0.053	0.707 ± 0.051	0.706 ± 0.048	0.717 ± 0.058	0.721 ± 0.052	4.279	0.001
	*p* value^b^	0.003	0.001	0.001	0.027	0.093		
DC	0.852 ± 0.031	0.829 ± 0.037	0.827 ± 0.036	0.827 ± 0.033	0.834 ± 0.041	0.837 ± 0.037	4.063	0.002
	*p* value^b^	0.004	0.002	0.001	0.030	0.106		
GMI	0.148 ± 0.053	0.159 ± 0.077	0.166 ± 0.082	0.186 ± 0.078	0.159 ± 0.069	0.159 ± 0.063	3.550	0.005
	*p* value^b^	0.683	0.228	0.001	0.662	0.658		
NTI	0.109 ± 0.068	0.132 ± 0.086	0.127 ± 0.076	0.107 ± 0.070	0.124 ± 0.089	0.120 ± 0.085	1.202	0.314

^a^Data were calculated by two-way analysis of variance (ANOVA)

^b^Data were calculated by Dunnett’s test to assess the difference between sdyn-eTHRIVE-CT and other MRI-CT

### Consistence of PTVs

The consistent parameters between CT and each MRI sequence for PTVs are shown in Table 5. Only the differences of GMI between different MRI sequence registrations to CT were significant (*p* < 0.05), and CT-T1, CT-T2, and CT-T2W-SPAIR were significantly different from CT-sdyn-eTHRIVE (all *p* < 0.05), respectively. Similarly, the overlap of sdyn-eTHRIVE to CT is the most in comparison with other MRI sequences to CT.

**Table 5:** Consistent parameters of registrations between CT and different MRI sequences in PTVs

Registrations	CT-sdyn-eTHRIVE	CT-T1	CT-T2	CT-T2W-SPAIR	CT-DWI	CT-dyn-eTHRIVE	*F*	*p* value^a^
CI	0.856 ± 0.039	0.836 ± 0.034	0.835 ± 0.032	0.839 ± 0.028	0.843 ± 0.039	0.844 ± 0.043	2.041	0.079
DC	0.922 ± 0.023	0.910 ± 0.021	0.910 ± 0.019	0.912 ± 0.017	0.914 ± 0.024	0.915 ± 0.026	1.903	0.100
GMI	0.051 ± 0.024	0.071 ± 0.042	0.073 ± 0.042	0.079 ± 0.045	0.062 ± 0.040	0.060 ± 0.034	6.661	2.000 × 10^−5^
	*p* value^b^	0.003	0.001	1.238 × 10^−5^	0.215	0.349		
NTI	0.093 ± 0.053	0.093 ± 0.054	0.091 ± 0.048	0.083 ± 0.048	0.096 ± 0.056	0.096 ± 0.063	0.566	0.726

^a^Data were calculated by two-way analysis of variance (ANOVA)

^b^Data were calculated by Dunnett’s test to assess the difference between sdyn-eTHRIVE-CT and other MRI-CT

### Correlation between clinical factors and CI

**Fig. 4:**
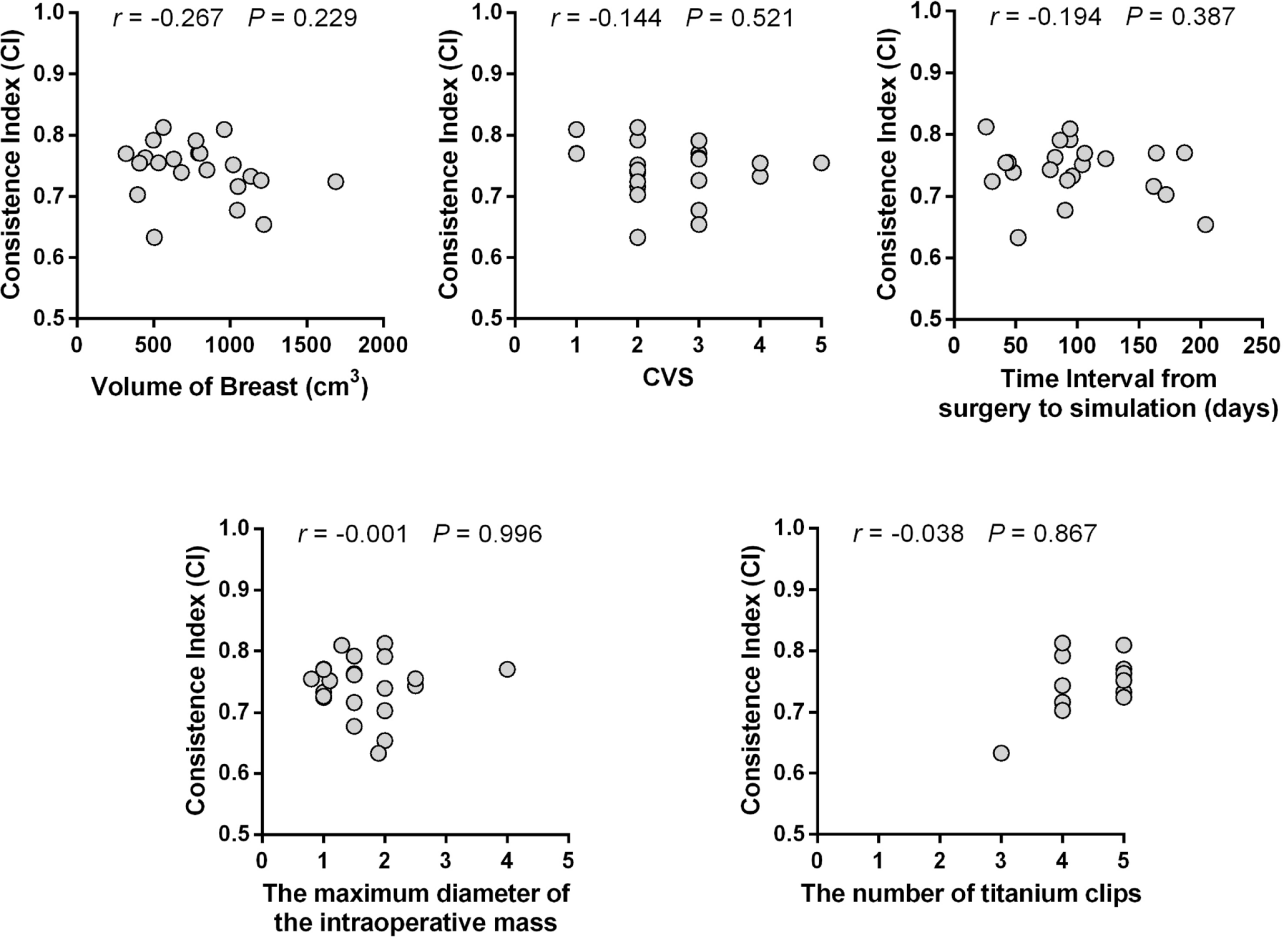
The correlation between the CI derived from the registration of CT and sdyn-eTHRIVE of CTV with the breast volume, the CVS of CT, the time interval from surgery to CT simulation, the maximum diameter of the intraoperative mass, and the number of titanium clips

We found that there is no obvious linear correlation (*p* > 0.05) between the CI derived from the registration of CT and sdyn-eTHRIVE of CTV with the breast volume, the CVS of CT, the time interval from surgery to CT simulation, the maximum diameter of the intraoperative mass, and the number of titanium clips, respectively (Fig. 4).

## Discussion

In this study, we compared the target volume of TB defined by pre-MRI and post-CT. The CTVs and PTVs in MRI sequences were all smaller than those in CT. The differences of delineation volumes among CT and MRIs were significant, both in the CTVs (*p* = 0.035) and PTVs (*p* < 0.001). And the values of CI and DC for sdyn-eTHRIVE registration to CT were the largest among all MRI sequences. We can conclude that CT overestimates the CTV and PTV on the assumption that preoperative MRI, and registering pre-MRI, especially sdyn-eTHRIVE, to post-CT could delineate the breast cancer more precisely.

Nowadays, whole-breast radiotherapy with boost to TB and accelerated partial breast irradiation (APBI) are two main radiotherapy regimens [22–26] and both of them require accurate definition of TB. Hence, many radiation oncologists have taken many measures to improve the current target delineation defined by the standard method—postoperative CT only—which could induce large inter-observer or intra-observer discrepancies with its poor soft resolution. Kirova et al have tried to register preoperative CT (pre-CT) to post-CT image associated with clips for better localization of the TB [27]. van der Leij et al have showed that the administration of pre-CT scans can decrease the inter-observer variation in the target volume delineation for external beam PBI [28]. As well, it is also verified by Boersma et al that combination with pre-CT could reduce inter-observer variation of post-CT-based boost TB delineation [29]. Nevertheless, the seroma and the LCs often shrink progressively with time [17, 30–32], and the clips may migrate out of the scope of postoperative TB [33] and easily be affected by any postoperative changes besides by re-excision. Even taking these two factors or combined with postoperative scar [30, 34] and preoperative imaging examinations into consideration at the same time, likewise, surgical clips are not always consistent with the edge of seroma [35]. With so many defects, these methods based on CT alone to definite the TB could lead to geographical miss or unnecessary normal tissue irradiation [36] and then may induce increased fibrosis.

In recent years, MRI is widely used in the screening, diagnosis, and treatment of breast cancer because of its good soft tissue resolution. It is superior to ultrasound and CT in determining the size, extension, and vascular relationship of the tumor, so it can more accurately define the TB range of breast cancer and reduce inter-observer or intra-observer variations [37–41]. T1-weighted MRI can better reflect the gross structural information and clips, while T2-weighted and dyn-eTHRIVE MRI can better reflect the situation of seroma [16]. Meanwhile, dyn-eTHRIVE MRI is also sensitive to the differences in micro-vessel density and vascular permeability, in turns to detect the visible or occult lesions more effectively [20, 42–45] and depict the TB more clearly [46], and subtraction imaging (sdyn-eTHRIVE) can improve the visualization of angiography and pathological contrast enhancement [20]. Our previous study demonstrated that post-MRI improved the CVS of post-CT, and the volumes generated based on MRI sequences are substantially smaller than those based on CT [16], the same with the result obtained by Giezen et al [18]. However, due to the poor capability to detect clips of post-MRI and postoperative seroma varies greatly with time, pre-MRI is also an effective alternative without appropriate time decided for post-MRI. As mentioned above, den Hartogh et al have found that the delineation of the TB on preoperative CT and MRI could decrease the target volume and the inter-observer target delineation compared with postoperative imaging [19, 47]. In this study, pre-MRI was registered to postoperative CT in order to determine the true location and the outline of the primary tumor, aiming to form a standard method for determining the TB. To be mentioned, all images of pre-MRI and post-CT were collected in prone position, which can keep all sequences consistent, maximize daily reproducibility, and improve the homogeneity of a breast plan [15, 48–50]. We also found that the volumes of pre-MRI were all smaller than CT-defined corresponding target volumes and there was a significant difference between sdyn-eTHRIVE and CT, further verifying the inaccuracy of the delineation only based on post-CT images. Furthermore, for both CTVs and PTVs, the overlap between the registrations of sdyn-eTHRIVE to CT is the largest. As for the value of the conformity parameters CI and DC in CTVs, each MRI registration to CT was significantly different (all *p* < 0.05) from CT-sdyn-eTHRIVE, except the CT-dyn-eTHRIVE, which could be explained by the fact that the images of sdyn-eTHRIVE were subtraction images from the dyn-eTHRIVE sequence. All these results suggested that the sdyn-eTHRIVE sequence of pre-MRIs did improve the delineation of CT-based CTV and PTV in comparison with other MRI sequences.

It is worth mentioning that, because optimal extent of conserving surgery with clear margins has not been established, on the basis of the clinical experience, the surgeon expanded 1.0 cm around the tumor after resection of the tumor lump to avoid peripheral residual occult lesions during surgery. So, we extended 1.0 cm from GTV to obtain the corresponding CTV, then expanded 1.5 cm from CTV to obtain the PTV, so MRI-based target volume was boosted by a 2.5-cm expansion from GTV. This method is similar to the study of van der Leij et al, who have reported that the CTV was obtained by expanding 2.0 cm from the GTV, then expanding 0.5 cm to obtain PTV, with a total 2.5-cm external margin from GTV to PTV [51]. In this study, according to rigorous and unified standard of registration, under the premise of maximizing the overlap of glandular tissue, other anatomical landmarks were kept as consistent as possible. On the account of this, the target volume of MRI was close to that of the CT, and the volume ratio approached 1.0, indicating that according to the surgeon’s clinical experience, extending 1.0 cm from GTV to obtain the corresponding CTV was practically significant, perhaps for further confirmation of pathological margins during surgery.

This study does contain some limitations. Firstly, because of the strict inclusion criteria, the sample size is obviously decreased. Secondly, the tumor location may lead to limitation, in that the border of CTV and PTV for tumors near the chest wall and skin is limited by the boundary. In particular, in comparison with CTV obtained by GTV expansion in MRI, the CTV of CT is based on the surgical cavity. Thus, the inconsistency of positioning the device between pre-MRI and post-CT, the deformation of breast after surgery, and the limitation of rigid registration itself could result in inevitable errors, which greatly increases the contingency of the results. Thirdly, because the volume of the postoperative cavity changes significantly with time and the time of CT localization is not defined clearly in this study, as a result, there are a great number of variabilities among delineations of different patients based on CT. Finally, the extended margin is derived from the clinical experience of surgeons, rather than from strict pathological confirmation. Thus, our results should be interpreted cautiously.

In summary, we used different sequences of pre-MRI registered to post-CT in order to guide the target volume delineation after breast-conserving surgery. In particular, when compared with other MRI sequences, sdyn-eTHRIVE might be a better choice to improve the delineation of CT-based CTV and PTV after breast-conserving surgery. This study provides ideas for further research about deformation registration, and the application of pre-MRI also lays a foundation of imaging to neoadjuvant radiotherapy. Further studies with a large sample size are needed to investigate the clinical application of pre-MRI images and post-CT images and establish a more precise strategy for postoperative radiotherapy based on MRI-CT images.

## Electronic supplementary material

ESM1 **Supplementary Figure 1.** Postoperative CT positioning device. **Supplementary Figure 2.** Preoperative MRI positioning device. (DOCX 58 kb)

## Abbreviations

APBI: Accelerated partial breast irradiation
CI: Consistence index
CT: Computed tomography
CTV: Clinical target volume
CVS: Cavity visualization score
DC: Dice coefficient
DWI: Diffusion-weighted imaging
dyn-eTHRIVE: Dynamic-enhanced T1 high-resolution isotropic volume excitation
GMI: Geographical miss index
GTV: Gross target volume
LCs: Lumpectomy cavity
MRI: Magnetic resonance imaging
NTI: Normal tissue index
post-CT: Postoperative computed tomography
post-MRI: Postoperative magnetic resonance imaging
pre-CT: Preoperative computed tomography
pre-MRI: Preoperative magnetic resonance imaging
PTV: Planning target volume
sdyn-eTHRIVE: Subtraction of dynamic-enhanced T1 high-resolution isotropic volume excitation
T2W-SPAIR: T2-weighted spectral attenuated inversion recovery
TB: Tumor bed
TE: Echo time
TR: Repetition time

## References

[1] Romestaing P, Lehingue Y, Carrie C (1997). Role of a 10-Gy boost in the conservative treatment of early breast cancer: results of a randomized clinical trial in Lyon, France. J Clin Oncol.

[2] Bartelink H, Horiot JC, Poortmans P (2001). Recurrence rates after treatment of breast cancer with standard radiotherapy with or without additional radiation. N Engl J Med.

[3] Bartelink H, Horiot JC, Poortmans PM (2007). Impact of a higher radiation dose on local control and survival in breast-conserving therapy of early breast cancer: 10-year results of the randomized boost versus no boost EORTC 22881-10882 trial. J Clin Oncol.

[4] Machtay M, Lanciano R, Hoffman J, Hanks GE (1994). Inaccuracies in using the lumpectomy scar for planning electron boosts in primary breast carcinoma. Int J Radiat Oncol Biol Phys.

[5] Krawczyk JJ, Engel B (1999). The importance of surgical clips for adequate tangential beam planning in breast conserving surgery and irradiation. Int J Radiat Oncol Biol Phys.

[6] Harrington KJ, Harrison M, Bayle P (1996). Surgical clips in planning the electron boost in breast cancer: a qualitative and quantitative evaluation. Int J Radiat Oncol Biol Phys.

[7] Mast M, Coerkamp E, Heijenbrok M (2014). Target volume delineation in breast conserving radiotherapy: are co-registered CT and MR images of added value?. Radiat Oncol.

[8] Landis DM, Luo W, Song J (2007). Variability among breast radiation oncologists in delineation of the postsurgical lumpectomy cavity. Int J Radiat Oncol Biol Phys.

[9] Li XA, Tai A, Arthur DW (2009). Variability of target and normal structure delineation for breast cancer radiotherapy: an RTOG multi-institutional and multiobserver study. Int J Radiat Oncol Biol Phys.

[10] Struikmans H, Warlam-Rodenhuis C, Stam T (2005). Interobserver variability of clinical target volume delineation of glandular breast tissue and of boost volume in tangential breast irradiation. Radiother Oncol.

[11] Whipp EC, Halliwell M (2008). Magnetic resonance imaging appearances in the postoperative breast: the clinical target volume-tumor and its relationship to the chest wall. Int J Radiat Oncol Biol Phys.

[12] Kirby AM, Yarnold JR, Evans PM (2009). Tumor bed delineation for partial breast and breast boost radiotherapy planned in the prone position: what does MRI add to X-ray CT localization of titanium clips placed in the excision cavity wall?. Int J Radiat Oncol Biol Phys.

[13] Sabine B, Giovanna D, Peter P, Clara J, Bert P, John K (2005). Open low-field magnetic resonance (MR) versus CT scanner (CT) imaging in breast radiotherapy treatment planning. Int J Radiat Oncol Biol Phys.

[14] Jolicoeur M, Racine ML, Trop I (2011). Localization of the surgical bed using supine magnetic resonance and computed tomography scan fusion for planification of breast interstitial brachytherapy. Radiother Oncol.

[15] Grann A, McCormick B, Chabner ES (2000). Prone breast radiotherapy in early-stage breast cancer: a preliminary analysis. Int J Radiat Oncol Biol Phys.

[16] Huang W, Currey A, Chen X (2016). A comparison of lumpectomy cavity delineations between use of magnetic resonance imaging and computed tomography acquired with patient in prone position for radiation therapy planning of breast cancer. Int J Radiat Oncol Biol Phys.

[17] Kader HA, Truong PT, Pai R (2008). When is CT-based postoperative seroma most useful to plan partial breast radiotherapy? Evaluation of clinical factors affecting seroma volume and clarity. Int J Radiat Oncol Biol Phys.

[18] Giezen M, Kouwenhoven E, Scholten AN (2012). MRI- versus CT-based volume delineation of lumpectomy cavity in supine position in breast-conserving therapy: an exploratory study. Int J Radiat Oncol Biol Phys.

[19] den Hartogh MD, Philippens ME, van Dam IE (2014). MRI and CT imaging for preoperative target volume delineation in breast-conserving therapy. Radiat Oncol.

[20] Gilles R, Guinebretiere JM, Toussaint C (1994). Locally advanced breast cancer: contrast-enhanced subtraction MR imaging of response to preoperative chemotherapy. Radiology.

[21] Smitt MC, Birdwell RL, Goffinet DR (2001). Breast electron boost planning: comparison of CT and US. Radiology.

[22] Julian TB, Costantino JP, Vicini FA et al (2011) OT2-06-02: a randomized phase III study of conventional whole breast irradiation (WBI) vs partial breast irradiation (PBI) for women with stage 0, 1, or 2 breast cancer: NSABP B-39/RTOG 0413. 10.1158/0008-5472.sabcs11-ot2-06-02:OT2-06-02-OT02-06-02

[23] Formenti SC, Truong MT, Goldberg JD (2004). Prone accelerated partial breast irradiation after breast-conserving surgery: preliminary clinical results and dose-volume histogram analysis. Int J Radiat Oncol Biol Phys.

[24] Taghian AG, Kozak KR, Doppke KP (2006). Initial dosimetric experience using simple three-dimensional conformal external-beam accelerated partial-breast irradiation. Int J Radiat Oncol Biol Phys.

[25] Smith TE, Lee D, Turner BC, Carter D, Haffty BG (2000). True recurrence vs. new primary ipsilateral breast tumor relapse: an analysis of clinical and pathologic differences and their implications in natural history, prognoses, and therapeutic management. Int J Radiat Oncol Biol Phys.

[26] Offersen BV, Overgaard M, Kroman N, Overgaard J (2009). Accelerated partial breast irradiation as part of breast conserving therapy of early breast carcinoma: a systematic review. Radiother Oncol.

[27] Kirova YM, Servois V, Reyal F, Peurien D, Fourquet A, Fournier-Bidoz N (2011). Use of deformable image fusion to allow better definition of tumor bed boost volume after oncoplastic breast surgery. Surg Oncol.

[28] van der Leij F, Elkhuizen PH, Janssen TM (2014). Target volume delineation in external beam partial breast irradiation: less inter-observer variation with preoperative- compared to postoperative delineation. Radiother Oncol.

[29] Boersma LJ, Janssen T, Elkhuizen PH (2012). Reducing interobserver variation of boost-CTV delineation in breast conserving radiation therapy using a pre-operative CT and delineation guidelines. Radiother Oncol.

[30] Hepel JT, Evans SB, Hiatt JR (2009). Planning the breast boost: comparison of three techniques and evolution of tumor bed during treatment. Int J Radiat Oncol Biol Phys.

[31] Wong EK, Truong PT, Kader HA (2006). Consistency in seroma contouring for partial breast radiotherapy: impact of guidelines. Int J Radiat Oncol Biol Phys.

[32] Weed DW, Yan D, Martinez AA, Vicini FA, Wilkinson TJ, Wong J (2004). The validity of surgical clips as a radiographic surrogate for the lumpectomy cavity in image-guided accelerated partial breast irradiation. Int J Radiat Oncol Biol Phys.

[33] Bernaerts ADSAJ, Van Dam P, Pouillon M (2007). Clip migration after vacuum-assisted stereotactic breast biopsy: a pitfall in preoperative wire localization. JBR-BTR.

[34] Oh KS, Kong FM, Griffith KA, Yanke B, Pierce LJ (2006). Planning the breast tumor bed boost: changes in the excision cavity volume and surgical scar location after breast-conserving surgery and whole-breast irradiation. Int J Radiat Oncol Biol Phys.

[35] Yang Z, Chen J, Hu W (2010). Planning the breast boost: how accurately do surgical clips represent the CT seroma?. Radiother Oncol.

[36] Landis DM, Luo W, Song J (2007). Variability among breast radiation oncologists in delineation of the postsurgical lumpectomy cavity. Int J Radiat Oncol Biol Phys.

[37] Aisen AM, Martel W, Braunstein EM, McMillin KI, Phillips WA, Kling TF (1986). MRI and CT evaluation of primary bone and soft-tissue tumors. AJR Am J Roentgenol.

[38] Petasnick JP, Turner DA, Charters JR, Gitelis S, Zacharias CE (1986). Soft-tissue masses of the locomotor system: comparison of MR imaging with CT. Radiology.

[39] Parker CC, Damyanovich A, Haycocks T, Haider M, Bayley A, Catton CN (2003) Magnetic resonance imaging in the radiation treatment planning of localized prostate cancer using intra-prostatic fiducial markers for computed tomography co-registration. Radiother Oncol 66:217–224 10.1016/s0167-8140(02)00407-3 12648794

[40] Mitchell DG, Snyder B, Coakley F (2006). Early invasive cervical cancer: tumor delineation by magnetic resonance imaging, computed tomography, and clinical examination, verified by pathologic results, in the ACRIN 6651/GOG 183 Intergroup Study. J Clin Oncol.

[41] Ireland RH, Bragg CM, McJury M (2007). Feasibility of image registration and intensity-modulated radiotherapy planning with hyperpolarized helium-3 magnetic resonance imaging for non-small-cell lung cancer. Int J Radiat Oncol Biol Phys.

[42] Liu PF, Debatin JF, Caduff RF, Kacl G, Garzoli E, Krestin GP (1998). Improved diagnostic accuracy in dynamic contrast enhanced MRI of the breast by combined quantitative and qualitative analysis. Br J Radiol.

[43] Orel SG, Schnall MD (2001). MR imaging of the breast for the detection, diagnosis, and staging of breast cancer. Radiology.

[44] Boetes C, Barentsz JO, Mus RD (1994). MR characterization of suspicious breast lesions with a gadolinium-enhanced TurboFLASH subtraction technique. Radiology.

[45] Heywang-Köbrunner SH, Bick U, Bradley WG (2001). International investigation of breast MRI: results of a multicentre study (11 sites) concerning diagnostic parameters for contrast-enhanced MRI based on 519 histopathologically correlated lesions. Eur Radiol.

[46] Wang CH, Yin FF, Horton J, Chang Z (2014). Review of treatment assessment using DCE-MRI in breast cancer radiation therapy. World J Methodol.

[47] den Hartogh MD, Philippens ME, van Dam IE (2015). Post-lumpectomy CT-guided tumor bed delineation for breast boost and partial breast irradiation: can additional pre- and postoperative imaging reduce interobserver variability?. Oncol Lett.

[48] Bieri S, Russo M, Rouzaud M, Kurtz JM (1997). Influence of modifications in breast irradiation technique on dose outside the treatment volume. Int J Radiat Oncol Biol Phys.

[49] Kirby AM, Evans PM, Donovan EM, Convery HM, Haviland JS, Yarnold JR (2010). Prone versus supine positioning for whole and partial-breast radiotherapy: a comparison of non-target tissue dosimetry. Radiother Oncol.

[50] Uematsu T, Yuen S, Kasami M, Uchida Y (2008). Comparison of magnetic resonance imaging, multidetector row computed tomography, ultrasonography, and mammography for tumor extension of breast cancer. Breast Cancer Res Treat.

[51] van der Leij F, Bosma SC, van de Vijver MJ (2015). First results of the preoperative accelerated partial breast irradiation (PAPBI) trial. Radiother Oncol.

